# Recommendations for conducting longitudinal studies with people who are justice involved

**DOI:** 10.1017/cts.2022.410

**Published:** 2022-06-01

**Authors:** Marley F. Fradley, Amanda Praseuth, Rachel L. Bearden, Mollee K. Steely Smith, Lisa Evans, Nickolas D. Zaller, Melissa J. Zielinski

**Affiliations:** 1 University of Arkansas for Medical Sciences, Little Rock, AR, USA; 2 University of Arkansas, Fayetteville, USA

**Keywords:** Research design, justice-involved populations, retention, longitudinal research, community-based follow-up methods

Crisis stabilization units (CSUs) serve justice-involved populations experiencing ongoing mental health and substance use disorders. In Arkansas, where our research team is located, CSUs function as a diversion to jail for individuals who come into contact with law enforcement while experiencing mental health crises. CSU guests receive respite care, pharmacological intervention, psychotherapy, and referrals to needed community resources [1,2]. The population served by CSUs experience profound and unique challenges (e.g., resource deprivation, housing insecurity, and poverty) which are compounded by criminal justice involvement and mental and behavioral health symptoms [2]. Empirical research is needed to fully understand the barriers to care experienced by this population so that policy-makers, practitioners, and other stakeholders can leverage this information to increase access to needed services, identify potential intervention points, and improve overall quality of life in an underserved and underrepresented population. However, there are many challenges to longitudinal research with justice-involved populations such as those served by CSUs.

In this paper, we offer a series of recommendations to improve study engagement, standardization, retention rates, and overall data quality in longitudinal research aiming to track justice-involved populations. Our recommendations stem from lessons learned while conducting a longitudinal cohort study of people who are discharged from a CSU. Participants are enrolled during their CSU admission and then complete five follow-up assessments over a one-year follow-up period. Considering the challenges experienced by this population – and the substantial need for research that evaluates health outcomes and service needs for justice-involved persons – translational researchers would benefit from guidance about the skills, strategies, and research infrastructure necessary to successfully engage and retain justice-involved participants in longitudinal research.

## Lessons Learned

Table 1 summarizes the challenges faced by our research team when conducting longitudinal data collection with individuals discharged from a CSU, the majority of whom were justice involved. The table also offers study design and training considerations for research with similar populations. It should be noted that the challenges observed in this population often intersect. For example, people experiencing substance use disorders may also experience increased admissions to residential treatment or hospitals and/or may be arrested or become incarcerated. Individually, these circumstances make it difficult to maintain contact and conduct follow-up assessments. When these challenges co-occur, they compound and create complex barriers to participant tracking and retention efforts. Thus, the recommendations outlined in Table 1 should be used concurrently and considered throughout all phases of research planning.

**Table 1.** Challenges and recommendations for longitudinal studies with justice-involved persons

Challenges	Recommendations
**Study Organization and Staff Training**	
Non-standardized training methods can contribute to poor retention.	Create a procedure manual which includes standardized methods for participant tracking and assessment administration. Continually refine and update as the study progresses. Outline expectations related to participant interactions, documentation, data collection, and data entry.
Participants are unlikely to have prior experience participating in research studies and at times may misunderstand the study goals.	Emphasize the study purpose and the participant’s role during the consent process. Reassess consent with participants as needed throughout the follow-up period and review the informed consent document if warranted. Build rapport through professionalism (e.g. maintaining objective neutrality, demonstrating mutual respect). Staff should continually reaffirm their role as an objective researcher at every contact with participants.
**Participant Retention and Engagement**	
Participants may have limited or frequently changing contact information, may be currently unhoused, or experiencing inconsistent housing.	Become familiar with local community organizations serving homeless populations and identify a point person at the organization to facilitate contact with participants. Post awareness flyers with study contact information at shelters or other locations that study participants might frequent. Be diligent when collecting contact information and obtain as many sources as possible. Consider alternative options for maintaining contact or determining whereabouts (e.g. healthcare providers, supervision officers, public libraries). Verify and update participant contact information fully at every opportunity. Ask participants to include community organizations that they are in contact with in their contact information. Be prepared to cold-call these organizations for help locating the participant when needed.
Maintaining contact is difficult for participants experiencing competing life obligations.	Establish a standard schedule for retention efforts. Reach out to participants multiple times and using multiple methods. Schedule staff coverage in a way that allows for “pop-up” assessments when participants who have been hard to reach indicate that they are available. Build correspondence templates for routine retention messages to be sent by text, email, social media, and physical mail to communicate with participants about upcoming appointments. Meet regularly to discuss hard-to-reach participants and collaboratively outline a plan for continued attempts.
Some participants may become incarcerated.	As a precaution, include approval to conduct assessments with incarcerated persons in the IRB protocol. The protocol should also include flexibility in assessment administration (e.g. by phone, mail, in-person, tele-video) to allow the team to adapt to facility policies. Familiarize the study staff with the local carceral system. Use publicly-available sources (e.g., court record databases, jail and prison rosters) to search for participants who you have been unable to contact. Identify a point-person at local probation/parole offices and mental health and drug courts to contact regarding participant incarceration status when needed. Take an individualized and collaborative approach to requesting permission to conduct research activities with a participant who is incarcerated.
Participants may experience periods of hospitalization, ongoing physical health problems, and unexpected injury/illness, which impact availability and limit study engagement.	Accommodate participant needs by offering alternate assessment administration methods (e.g. emailing assessment PDFs so participants can follow along, taking frequent breaks, mailing physical copies for self-complete). Communicate with social support networks (e.g. family, friends, health providers) to remain updated on the participant’s health status.
**Participant Interactions**	
Many participants may have untreated or ongoing mental health conditions such as depression, anxiety, PTSD, and psychosis. At times, they may endorse suicidal ideation or reach out to study staff in active crisis.	Develop a protocol and screener for identifying and appropriately responding to suicidal ideation. Train staff to administer screener and document screener outcome. Identify a licensed mental health provider who can consult with study staff, make recommendations, and potentially step-in to ensure participant safety during crises. Educate staff on crisis resources and procedure for conducting emergency welfare checks.
Active drug or alcohol use during follow-up assessments may reduce participant capacity for comprehension and recall, increase sensitivity to assessment topics, and may create issues with data quality and validity.	Discontinue or reschedule follow-up assessments if participation is impaired by acute intoxication. Be prepared to refer participants to local substance use resources or agencies that can provide these referrals.
Some participants may become distressed or feel uncomfortable when answering questions related to mental health symptoms, trauma, criminal-justice history, and drug and alcohol use.	Validate participant experiences and express appreciation for willingness to share. Encourage participants to take breaks as needed to avoid distress. Avoid responses that indicate positive or negative judgment during assessments (e.g. “I’m glad to hear that.” or “That’s terrible!”). Remain neutral during assessment administration to normalize participant experiences and avoid participant response bias.
Assessment length can result in participant fatigue.	Check in with participants throughout assessments and offer breaks. Be aware of response incongruity as a potential indicator of fatigue. Offer multiple methods for completing assessments when needed.
**Unprecedented Circumstances**	
Global crises (e.g. pandemics) can impact study progress including, recruitment enrollment, retention efforts, and assessment administration.	Plan for an increased timeline and, if conducting grant-funded research, consider a no-cost extension. Be flexible and willing to implement novel approaches to accomplish study goals, such as increased options for remote participation.
Internal and external staff changes can result in poor adherence to study procedures and lost contact with community partners.	Cross-train study staff in all aspects of study procedure. Ensure each role has a designated backup. Meet regularly to discuss study updates and follow-up efforts. Staff who leave the study should ensure that remaining staff absorb their responsibilities before exiting. Avoid identifying a single contact person for a community organization. Identify a backup for this person or establish a relationship with the organization’s leadership.

Additionally, each participant and the unique challenges they may experience should be evaluated to determine which considerations will be most relevant. Strategies that work well for some participants may not work for others, even if they appear to be experiencing the same challenges. Documentation of all contact attempts and retention efforts is essential to identifying variables that contribute to reduced study engagement and can help study staff determine which strategies have successfully mitigated challenges in the past.

## Conclusions

Conducting rigorous research with justice-involved populations requires forethought and flexibility from investigators. It is important to mirror the needed flexibility in the study protocol—particularly with regard to the methods and resources used to remain in contact with participants—and to adequately communicate the benefits and risks of enrolling in a longitudinal study within the informed consent document(s). The study from which these recommendations were formed was methodically planned out and rigorously designed; however, unprecedented challenges (e.g., the COVID-19 pandemic) required study staff to continually reevaluate study procedures and revise training manuals. This resulted in the development of briefing documents, training presentations, and manuals which ensured that all study staff received standardized training and were able to handle unexpected challenges. Table 2 describes these materials. We also purchased access to a web-based, HIPPA-compliant participant management and scheduling tool [3] which facilitated documentation of all participant contact attempts and history of study engagement.

**Table 2.** Training and procedural materials

Document	Purpose
Manual	A detailed and comprehensive guide outlining all study procedures
Study Overview and Training Presentation	A presentation designed to orient new team members to the project objectives and procedures
Participant Interactions Training Presentation	A presentation created to train staff on how to appropriately and effectively communicate with diverse participants, including tips on how to proceed in challenging circumstances
Suicidality Screener and Protocol	A detailed protocol adapted from the Columbia-Suicide Severity Rating Scale for identifying and screening for suicidality and promoting participant safety
Welfare Check Training	A guide on how to initiate and document an emergency welfare check in the event a participant discloses imminent intent to take actions to end their own life.
Community Resource Guides	A guide describing local community resources that may be beneficial to participants

As evidenced in Table 1, most challenges within the current study centered on maintaining regular contact with study participants and completing follow-up assessments. Though data collection is ongoing, retention rates have remained relatively stable—near 75% for most time-points. This is in part because study staff cultivated relationships with community organizations who also serve the study population. To date, study staff have worked with 45 organizations, including residential treatment facilities, assisted living facilities, homeless shelters, community outreach organizations, day centers, mental health clinics, and correctional settings to maintain contact with participants. The ability to successfully engage these agencies has been key to contacting participants and keeping them engaged with the study. These successes demonstrate the efficacy of the recommendations described in Table 1. We hope the lessons we learned in conducting health research with justice-involved individuals will be considered when designing future longitudinal research studies with similar populations.
